# Occupational allergic contact dermatitis caused by colophonium, an unsuspected sensitizer in a petrochemical worker

**DOI:** 10.1111/cod.14211

**Published:** 2022-09-17

**Authors:** Sami Hamwi, Antoine Kunst, Christine Boust, Olivia Bauvin, Florence Tetart

**Affiliations:** ^1^ Department of Dermatologie CHU de Rouen Rouen France; ^2^ CARSAT Normandie Rouen France

Colophonium is a common cause of (occupational) contact allergy. The present case illustrates occupational airborne allergic contact dermatitis from this sensitizer in a rather unexpected context, that is, in a petrochemical industrial environment.

## CASE REPORT

**FIGURE 1:**
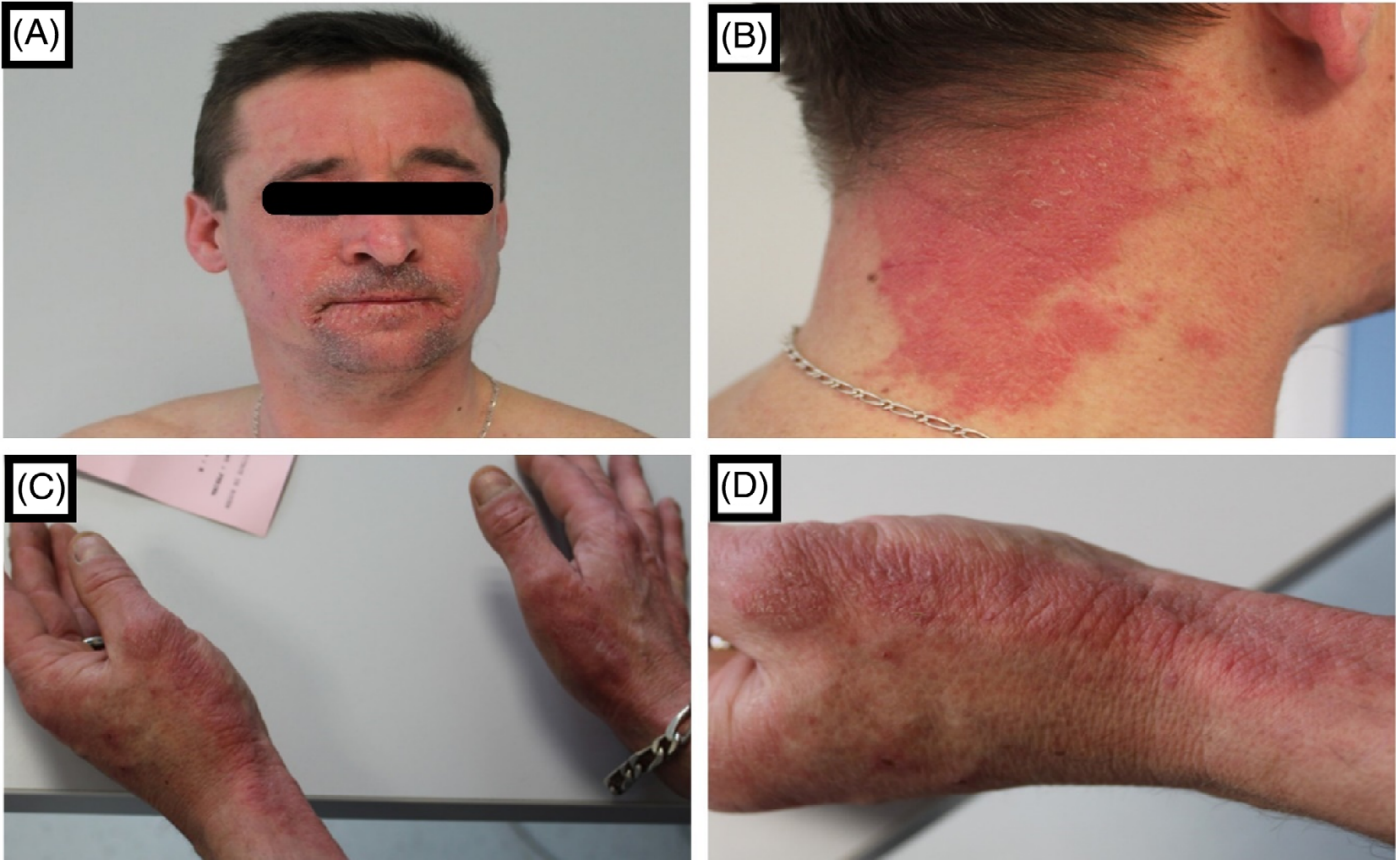
(A–D) The patient with an eczematous rash predominantly on his face, neck and upper limbs/hands

**FIGURE 2:**
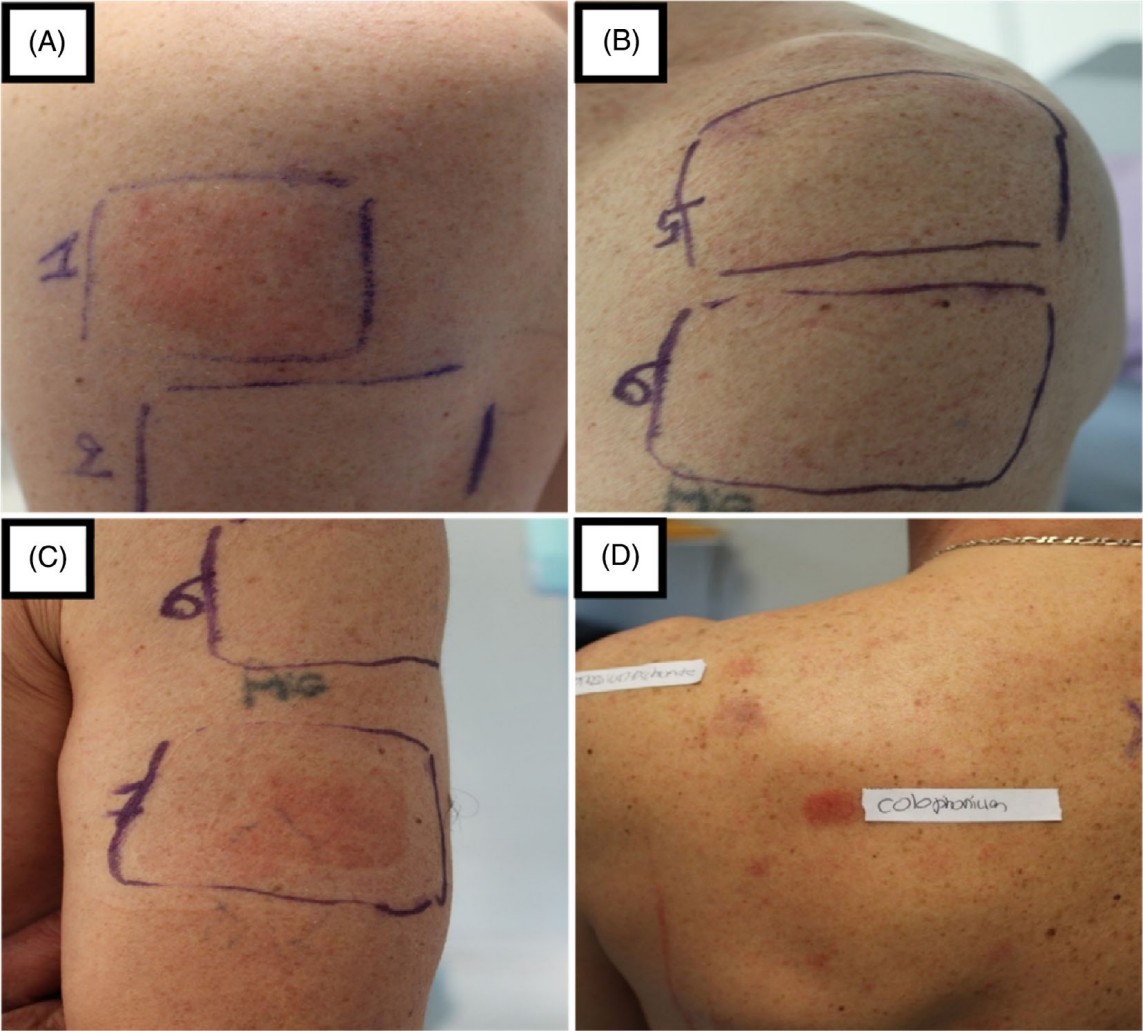
(A) Semi‐open test (+) with wood dust humidified with saline (1). (B) Semi‐open test (negative) with unused gloves, inside (5) and outside (6). (C) Semi‐open test with used gloves, inside (6; negative) and outside (7; +). (D) Patch test positive (++) for colophonium at D3.

A 48‐year‐old man, who had suffered from asthma as a child, was referred for the evaluation of a pruritic eczematous rash on his hands, that subsequently also affected his face and neck (Figure 1). The skin lesions had developed several months after he had started working as a forklift operator on a packaging line for a company that manufactures petrochemical derivatives. His job (whilst usually wearing gloves) consisted in three tasks: packing rubber materials (“blue polymers” and “white rubber”) with plastic film; production and assembling of wooden crates which contain these rubber materials; and moving the crates with a forklift. Patch tests were performed with the European Comprehensive Baseline series, a rubber series, an in‐house glove series, and a (meth)acrylates series (haptens and patch test chambers from Chemotechnique Diagnostics, Vellinge, Sweden). Semi‐open tests were performed with humidified dust of the “blue polymers”, the “white rubber”, and the used gloves. A small piece of the “blue polymer” and of the “white rubber” were crushed to dust and humidified with sterile water. A small amount of this suspension was then applied with a cotton swab onto the skin, left to dry, and covered with acrylate tape. Following an occlusion of 2 days readings were performed on day (D) 2 and D3, according to criteria of the International Contact Dermatitis Research Group, and showed positive reactions to chromium (+) and colophonium (++); the semi‐open tests were only positive for the outside of the used gloves (Figure 2). No relevance was found for chromium. After repeated questioning, the patient informed us he worked in a wood‐dust environment, and that he himself manufactured the wooden crates by cutting and assembling the wood. Allergic contact dermatitis caused by a component of wood dust, such as colophonium, was then highly suspected, and a second test session was performed. Additional semi‐open tests with wood dust (from the wooden crates) were positive, whereas semi‐open tests with the inside and outside of a pair of identical (yet unused) gloves remained negative, confirming that the previously observed positive semi‐open tests with the outside of the used gloves were due to contamination with wood dust. Chemical analyses of the wood dust samples, by means of gas chromatography and Fourier transform infrared spectroscopy, identified the presence of colophonium in the wood dust, and thus confirmed this contact allergen as a relevant and occupational sensitizer. After discussing the case with the occupational physician, the patient was moved to a post without any exposure to wood dust, following which significant improvement of his skin condition occurred. The patient has given written consent to publish data and photographs.

## DISCUSSION

The prevalence of colophonium allergy is 0.7% in an unselected population, and 3% in a population that had an episode of eczema the previous year.^1^ Colophonium is a complex mixture containing >100 compounds derived from pine trees. It has countless applications at home,^2^ and also in the work environment, and exposure to (oxidation products of) colophonium, and also to modified colophonium, is ubiquitous. We highlight this case as colophonium exposure is rather unexpected in a petrochemical industrial environment.^3^, ^4^, ^5^, ^6^ Querying the French database of occupational exposure (SOLVEX^4^) with the activity sector “chemical industry” and the post “storage and transport of materials” retrieved mainly aromatic compounds (“‐arene”), aldehydes and acetylene black. Although colophonium is also an ubiquitous sensitizer in daily life, our patient reported no hobby or activity that made us suspect sensitization in private life. Furthermore, the timing after which the patient developed skin lesion rather favoured an (unexpected) occupational origin.

## CONFLICT OF INTEREST

The authors declare no conflict of interest.
